# Harliku (nitisinone): first FDA-approved disease-modifying therapy for alkaptonuria

**DOI:** 10.1097/MS9.0000000000004033

**Published:** 2025-10-06

**Authors:** Harshika Khaim Chandani, Syeda Fadak Zahra Hujjat, Devya Khaim Chandani, Muhammad Saad Khan, Abubakr Mahmoud

**Affiliations:** a Department of Medicine, Jinnah Sindh Medical University, Karachi, Pakistan; b Allama Iqbal Medical College, Lahore, Pakistan; c Department of Medicine, Shaheed Mohtarma Benazir Bhutto Medical College (SMBBMC), Karachi, Pakistan; d Department of Medicine, University of Khartoum, Khartoum State, Sudan

**Keywords:** alkaptonuria, disease-modifying therapy, Harliku, homogentisic acid, nitisinone, ochronosis

## Abstract

Alkaptonuria (AKU) is a rare autosomal recessive metabolic disorder due to a deficiency of homogentisate 1,2-dioxygenase, subsequently leading to the progressive acquisition of ochronosis, osteoarthropathy, systemic complications, and the pathological retention of homogentisic acid (HGA). Until recently, apart from supportive management which involved orthopedic surgery and symptomatic treatment, there was no attempt to slow the progress of the disease. On 19 June 2025, the U.S. Food and Drug Administration approved Harliku (nitisinone) as the first therapy to modify the progression of the disease. As a competitive inhibitor of 4-hydroxyphenylpyruvate dioxygenase, nitisinone exerts biochemical efficacy with a greater than 95% reduction of urinary excretion of HGA. The SONIA 2 and other studies funded by the NIH have demonstrated long-standing metabolic control with nitisinone, as well as improved functional outcomes and possibly cardioprotective effects. Although the evidence of long-term benefits is promising, risks of treatment, such as tyrosinemia, require attention to diet and continued monitoring. As the first single agent to be taken orally once daily, Harliku changes the paradigm of management for the rare metabolic disorder AKU from symptomatic treatment to disease-modifying therapy. It is a huge leap for other disorders of metabolism and paves the way for future innovations in therapy.

## Background

Alkaptonuria (AKU) is a rare autosomal recessive metabolic disease resulting from mutations in the HGD gene, which codes for homogentisate 1,2-dioxygenase. This enzyme deficiency prevents tyrosine and phenylalanine from being catabolized normally, which causes homogentisic acid (HGA) to build up in tissues and urine. This causes early-onset osteoarthritis, ochronosis of cartilage, dark urine, and progressive problems in the kidneys and heart^[1]^. About 1 in 100 000–250 000 people worldwide suffer from AKU, which causes significant musculoskeletal morbidity, chronic pain, functional deterioration, and a lower quality of life^[2]^. This manuscript has been developed in accordance with the TITAN Guidelines (2025)^[3]^.

Up until recently, the only supportive measures used to treat AKU were joint replacement, pain management, and the treatment of complex aftereffects. With respect to this, the first and only FDA-licensed medication for this extremely rare ailment, Harliku (nitisinone) pills were approved by the U.S. Food and Drug Administration on 19 June 2025, for the lowering of urine HGA in adult patients with AKU^[4]^. Nitisinone is a strong competitive 4-hydroxyphenylpyruvate dioxygenase (HPPD) inhibitor. It lowers the abnormal accumulation by blocking HPPD, which completely stops homogentisic acid from forming. By blocking the tyrosine degradation pathway upstream, this approach differs from earlier supportive management strategies in that it directly treats the main metabolic problem rather than just symptom relief^[5]^.

**HIGHLIGHTS**

- **FDA approval of Enflonsia™ (clesrovimab-cfor)** introduces a single-dose, season-long prophylaxis for neonatal respiratory syncytial virus (RSV) prevention.
- **High-affinity targeting of prefusion RSV F protein** ensures potent neutralization against both RSV A and B strains.
- **Clinical trials (MELODY, MEDLEY, Phase 2b)** demonstrated up to 80% reduction in RSV-related hospitalizations with a favorable safety profile.
- **Once-per-season intramuscular dosing** offers logistical advantages over multi-dose regimens like palivizumab.
- **Potential global health impact** through reduced RSV burden, though accessibility and cost remain key challenges.

## Mechanism of action and pharmacokinetics

An enzyme upstream of HGD called HPPD is strongly competitively inhibited by nitisinone. Nitisinone reduces pathological buildup and stops the cascade that leads to ochronosis by inhibiting HPPD activity, which stops homogentisic acid from forming^[5]^. Nitisinone exhibits a high oral bioavailability (>95%) in terms of pharmacokinetics, guaranteeing consistent systemic exposure. Three hours after the treatment, its peak plasma concentrations are attained. Once-daily dosing is possible due to its lengthy elimination half-life (~50–60 hours)^[6]^. It is mostly metabolized in the liver, with inactive metabolites being cleared by the kidneys. But because metabolism is diverted, plasma tyrosine levels rise sharply, frequently surpassing 600 µmol/L. Regular monitoring is essential since ophthalmic and dermatological problems are caused by this hyper-tyrosinemia^[7]^.

## Dosing and administration

The recommended adult dose for AKU is 10 mg orally once daily, which achieves a >95% reduction in urinary HGA^[8]^. Tablets can be taken with or without food. Clinical use mandates routine monitoring of plasma tyrosine levels, liver function testing to identify hepatotoxicity, ophthalmologic evaluation for corneal opacities or keratitis, and dietary restriction of tyrosine and phenylalanine, guided by metabolic nutritionists to minimize complications. Nitisinone’s once-daily oral regimen offers excellent adherence potential compared to invasive surgical or supportive interventions.

## Evaluation of safety and efficacy through clinical trials

The clinical usefulness of nitisinone in AKU has been demonstrated by numerous trials. Over the course of 4 years, 138 AKU patients participated in the big, worldwide, open-label, randomized SONIA 2 trial, which compared nitisinone 10 mg daily with no therapy. Strong biochemical efficacy was confirmed by a 99% decrease in urine HGA caused by nitisinone. However, structural progression of ochronosis and joint damage was only modestly slowed, highlighting the need for long-term follow-up^[9]^. An NIH-led long-term randomized trial was done, which was a 3-year, no-treatment controlled study of 40 AKU patients (20 receiving 2 mg daily nitisinone plus standard care vs. 20 controls). The treated patients experienced improvements in pain, energy, and physical functioning as measured by SF-36 and the 6-minute walk test despite the primary endpoint (hip range of motion) not reaching statistical significance. This analysis of the above NIH trial revealed sustained quality-of-life and functional gains over 3 years, reinforcing the real-world benefits of nitisinone therapy in AKU^[10]^. Moreover, in an 18-month NIH observational cohort study on cardiac progression, no nitisinone-treated AKU patients without baseline aortic sclerosis progressed to aortic sclerosis or stenosis, whereas 7 of 17 untreated controls did, suggesting a possible cardioprotective effect^[11]^. Collectively, these trials highlight the biochemical success, functional benefits, and potential systemic protection offered by nitisinone.

## Comparative perspective

Without nitisinone, AKU patients benefitted little from supportive care, analgesics, and surgical orthopedic interventions. It is disease-modifying and, in comparison, supportive metabolic barrier interventions remain in the realm of theory^[12]^. Gene therapy and enzyme replacement technology are emerging for rare, inborn metabolic disorders. Biochemical effects, albeit indirect, make it preferable over other non-invasive, oral, and readily available alternatives. The FDA approval makes it the benchmark for other novel therapies for inborn errors of metabolism.

## Complications

Most complications associated with nitisinone focus on tyrosinemic effects. Ocular, such as keratitis and corneal crystals, opacity, and photophobia, dermatologic, palmoplantar keratoderma, hematologic, such as leukopenia and thrombocytopenia, and hepatic, rare case reports of transaminitis and uncontrolled hyperammonemia transaminitis, remain unreasonable^[12]^. Most paternalistic side effects are manageable with dose alteration and tailored dietary interventions, require which prudent monitoring.

## Cost-effectiveness and future implications

Due to the scarcity of AKU, nitisinone will likely have orphan drug prices. Even when factoring the sizable cost, improving quality of life, reducing cardiovascular events, and delaying joint replacement surgery are other long-term health expenses that AKU accounts for^[13]^. There are also other methods that should be explored to gain a better hold of the disease, such as, the safety of the drug in question and its dosage strategies aided by other combinations to achieve a better efficacious control of tyrosinemia and gene or enzyme replacement therapies. There is also a need to have a better understanding of the role of the drug in children and its combination therapies^[7]^.

## Limitations and contraindications

Even though the drug is FDA approved, there are still unaddressed issues. In the case of nitisinone, which clinically prevents further damage, it is still unable to eradicate the damage that pre-exists the pigmentation. Functional results, or outcome measurements, are also rather modest, and that is primarily due to the nitisinone rational effectiveness. It still has uncertainties that have yet to be answered, which also surround its long-term safety (and in this case, specifically ocular toxicity). Known hypersensitivity to nitisinone, inability to monitor and control, and also severe hepatic impairment are included within the contraindications.

## Conclusion

In summary, Harliku (nitisinone) represents a landmark in the management of AKU, being the first FDA-approved therapy that targets the root metabolic defect rather than symptoms. By dramatically reducing urinary homogentisic acid and showing functional and possible cardioprotective benefits, it ushers in a new era of disease modification for ultra-rare metabolic disorders. It enhances functional outcomes and prevents or stops the progression of problems. Its once-daily oral regimen improves adherence and feasibility. However, vigilance is required for tyrosine-related complications, cost accessibility, and long-term outcomes. As research advances, integrating pharmacological, dietary, and genetic therapies may eventually transform AKU from a relentlessly progressive disease into a manageable condition with preserved quality of life.

## Data Availability

No data were generated for this manuscript.

## Research registration unique identifying number (UIN)

Name of the registry: N/A. Unique Identifying number or registration ID: N/A. Hyperlink to your specific registration (must be publicly accessible and will be checked): N/A.
